# Growing Old Too Early: Skeletal Muscle Single Fiber Biomechanics in Ageing R349P Desmin Knock-in Mice Using the *MyoRobot* Technology

**DOI:** 10.3390/ijms21155501

**Published:** 2020-07-31

**Authors:** Charlotte Pollmann, Michael Haug, Barbara Reischl, Gerhard Prölß, Thorsten Pöschel, Stefan J Rupitsch, Christoph S Clemen, Rolf Schröder, Oliver Friedrich

**Affiliations:** 1 Institute of Medical Biotechnology, Friedrich-Alexander-University Erlangen-Nürnberg, Paul-Gordan-Str. 3, 91052 Erlangen, Bavaria, Germany; charlotte.pollmann@fau.de (C.P.); barbara.reischl@fau.de (B.R.); g.proelss@fau.de (G.P.); oliver.friedrich@fau.de (O.F.); 2 Graduate School in Advanced Optical Technologies, Paul-Gordan-Str. 6, 91052 Erlangen, Bavaria, Germany; 3 School of Medical Sciences, University of New South Wales, Wallace Wurth Building, 18 High St, Sydney, NSW 2052, Australia; 4 Institute of Multi Scale Simulation of Particulate Systems, Friedrich-Alexander-University Erlangen-Nürnberg, Nägelbachstr. 49b, 91052 Erlangen, Bavaria, Germany; thorsten.poeschel@fau.de; 5 Institute of Sensor Technology, Friedrich-Alexander-University Erlangen-Nürnberg, Paul-Gordan-Str. 3/5, 91052 Erlangen, Bavaria, Germany; stefan.rupitsch@fau.de; 6 German Aerospace Center (DLR), Institute of Aerospace Medicine, Linder Höhe, 51147 Cologne, North Rhine-Westphalia, Germany; christoph.clemen@uni-koeln.de; 7 Institute of Neuropathology, University Hospital Erlangen, Friedrich-Alexander University Erlangen-Nürnberg, Schwabachanlage 6, 91054 Erlangen, Bavaria, Germany; rolf.schroeder@uk-erlangen.de; 8 Insitute of Vegetative Physiology, Medical Faculty, University of Cologne, Center of Physiology and Pathophysiology, Robert-Koch-Street 39, 50931 Cologne, North Rhine-Westphalia, Germany; 9 Muscle Research Center Erlangen (MURCE), Friedrich-Alexander-University Erlangen-Nürnberg, 91054 Erlangen, Bavaria, Germany; 10 Victor Chang Cardiac Research Institute, Lowy Packer Building, 405 Liverpool St, Sydney, NSW 2010, Australia; 11 Optical Imaging Centre Erlangen OICE, Cauerstr. 3, 91058 Erlangen, Bavaria, Germany

**Keywords:** biomechatronics, desminopathy, R349P desmin, single fibers, skeletal muscle

## Abstract

Muscle biomechanics relies on active motor protein assembly and passive strain transmission through cytoskeletal structures. The desmin filament network aligns myofibrils at the z-discs, provides nuclear–sarcolemmal anchorage and may also serve as memory for muscle repositioning following large strains. Our previous analyses of R349P desmin knock-in mice, an animal model for the human R350P desminopathy, already depicted pre-clinical changes in myofibrillar arrangement and increased fiber bundle stiffness. As the effect of R349P desmin on axial biomechanics in fully differentiated single muscle fibers is unknown, we used our *MyoRobot* to compare passive visco-elasticity and active contractile biomechanics in single fibers from fast- and slow-twitch muscles from adult to senile mice, hetero- or homozygous for the R349P desmin mutation with wild type littermates. We demonstrate that R349P desmin presence predominantly increased axial stiffness in both muscle types with a pre-aged phenotype over wild type fibers. Axial viscosity and Ca${}^{2+}$-mediated force were largely unaffected. Mutant single fibers showed tendencies towards faster unloaded shortening over wild type fibers. Effects of aging seen in the wild type appeared earlier in the mutant desmin fibers. Our single-fiber experiments, free of extracellular matrix, suggest that compromised muscle biomechanics is not exclusively attributed to fibrosis but also originates from an impaired intermediate filament network.

## 1. Introduction

Skeletal muscle is the largest organ system of the body and under constant mechanical axial and lateral stress, either due to passive strain or through active contraction. While lateral forces are distributed between single fibers across anchorage points in the extracellular matrix (ECM) to the intracellular cytoskeleton via the dystrophin–glycoprotein complex [1] and focal adhesion complexes [2], axial forces are distributed through contractile (active) and non-contractile (passive) elements. Apart from the giant, approximately 1.5 μm long elastomeric protein titin being responsible for the visco-elastic properties of single muscle fibers through unfolding of globular domains under strain [3,4], the connecting proteins of the extra-sarcomeric intermediate filament (IF) family are also a vital determinant of axial stiffness. An important member of the IFs is the type III filament protein desmin, transversely linking adjacent myofibrils at the level of the z-disc, and thus being responsible for the myofibrillar register [5,6,7]. In humans, desmin is encoded on chromosome 2q35 by a single-copy gene. The 53 kDa desmin presents a tripartite structure with a central-helical coiled-coil domain flanked by non-helical tail and head domains. Due to its intrinsic self-assembling properties, it builds three-dimensional networks, starting with supercoil formation via dimerization of two desmin molecules. Two such dimers then associate into tetramers that represent the repetitive add-on units for spontaneous assembly to 60 nm long filaments, the so-called unit-length filaments (ULFs [8]). Serial longitudinal annealing of ULFs consequently builds short filaments, extending the IF network. Eventually, long filaments reduce their diameter by spontaneous radial compaction to form the mature IF network. This network connects to multiple intracellular adhesion sites by cross-bridging proteins from the spectrin superfamily, i.e., plectin and nesprins [9].

In skeletal muscle, IFs form a huge stress-transmitting and stress-signaling network, in which desmin is important to maintain myofibrillar alignment, nuclear positioning and shape, stress production, and sensing [7,10]. Due to molecular state transitions (e.g., conversion from alpha helix to beta sheet) and subunit sliding capabilities of IF proteins, the IF network remains largely intact even when exposed to large physical strains, e.g., surviving at least 250% strains before rupture [11,12]. This led to their proposed role of acting as a cytoskeletal “position-memory” that ensures proper reassembly of cytoskeletal components after large strains [13]. The deleterious effects of abnormal desmin IF networks, due to either the additional presence of mutant or the complete lack of wild type desmin protein, are emphasized by the group of human desminopathies that comprise autosomal-dominant and recessively inherited myopathies and cardiomyopathies [8]. Human desminopathies are clinically characterized by a broad phenotypic variability ranging from primary distal myopathies, limb girdle muscular dystrophies, and scapuloperoneal syndromes to generalized myopathies [14,15,16,17]. The major problem with elucidating the pathophysiological mechanisms of human phenotypes is that knowledge about early and intermediate disease stages is usually elusive, as muscle tissue specimen are not available from patients at pre-clinical stages. Therefore, a patient-mimicking knock-in mouse strain carrying the R349P desmin mutation, the murine orthologous of the human R350P mutation, was generated [18]. This model already allowed detailed systematic studies of clinical and myopathological phenotypes as well as age-dependent effects on the disease progression in heterozygous (het) and homozygous (hom) desminopathy mice over their wild type (wt) littermates [19].

Our previous work already demonstrated that the expression of R349P mutated desmin compromises the 3D arrangement and the order of the myofibrillar lattice in young mice. These findings suggested a pre-aged phenotype of muscle structural aging in the R349P environment [20]. Moreover, biomechanical analyses of small fiber bundles, initially in slow-twitch, load-bearing *Musculus soleus* (SOL) fiber bundles from young het and hom R349P desmin mice, showed a marked increase in passive stiffness compared to wt bundles [18,19]. Consequently, we extended these previous recordings in SOL bundles from R349P desmin mice to fast-twitch *Musculus extensor digitorum longus* (EDL) and also included a wide age range from young (17–23 weeks) to aged (60–80 weeks) animals [21]. Again, the increased fiber bundle stiffness in young animals was confirmed in both muscle entities with a pre-aged phenotype in the desminopathy model. However, as ECM remodeling corroborates with increased levels of tissue fibrosis with age in the R349P background [19], the increased axial stiffness could not be exclusively/mostly attributed to the disrupted desmin network, but might still arise from enlarged amounts of fibrotic collagenous tissue. To tackle the idea of desmin-induced fiber stiffening in the R349P desminopathy model and its influence on force generating capabilities, the present study advances to single muscle fibers, virtually free from ECM influence or surrounding connective tissue. Unfortunately, single-cell biomechanics function recordings are very delicate and require precise actuation, e.g., to assess the fiber’s purely elastic properties in resting length–tension (RLT) curves or unloaded speed of shortening in “slack tests”. To provide a robust and reliable metrology for single muscle fiber biomechanics, we used our engineered *MyoRobot* biomechatronics system which features sensitive force transducer (FT) technology, high precision voice coil (VC) actuation, and automated chemical solution exchange to capture disease-related influences on active and passive forces [22]. Here, we provide new insights into (i) the connection of mutated desmin to axial active/passive biomechanics in single fibers and (ii) the age-dependent progression of altered fiber mechanics in the R349P desminopathy model.

## 2. Results

### 2.1. Ca${}^{2+}$-Mediated Force and Ca${}^{2+}$ Sensitivity in Single Fibers from R349P Desminopathy SOL and EDL Muscles at Different Ages

**Figure 1:**
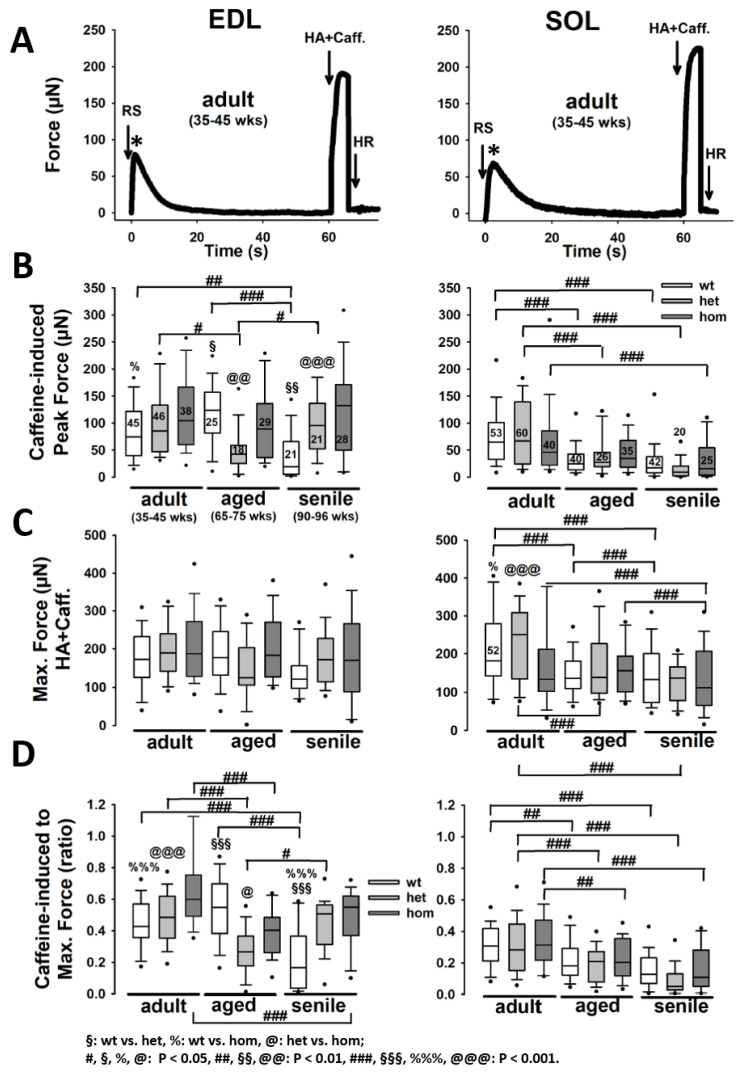
Caffeine-induced force and maximum Ca${}^{2+}$-saturated force in single *Musculus extensor digitorum longus* (EDL) and *Musculus soleus* (SOL) fibers from adult, aged, and senile R349P desmin mice. (**A**) Representative force recordings in a single EDL (left) and SOL (right) fiber. Group analysis of peak force amplitude during caffeine release (RS) (**B**), steady-state maximum force (HA) (**C**), and respective RS:HA force ratios (**D**) indicates an overall decrease in SR Ca${}^{2+}$ release force during aging in EDL and SOL, regardless of genotype. Within age groups, RS peak force was significantly larger in hom EDL fibers for the adult and senile groups, while they were similar in SOL. In EDL, there was no difference in maximum attainable force among genotypes regardless of age. Thus, RS:HA force ratios in EDL reflect the pattern differences of RS peaks, while in SOL fibers, relative force during SR Ca${}^{2+}$ release over maximum Ca${}^{2+}$-saturated forces were similar among genotypes and showed a significant decrease with age. Significance tested with two-way ANOVA followed by post hoc analysis (Bonferroni). Numbers in box plots: number of single fibers analyzed; also valid for panels (**C**,**D**). *: indicates caffeine-induced force maximum.

Figure 1A shows representative *MyoRobot*-recorded force transients of a caffeine-triggered Ca${}^{2+}$-mediated force response, which empties the sarcoplasmic reticulum (SR) of its releasable Ca${}^{2+}$ pool, followed by a maximum Ca${}^{2+}$-saturated activation of the contractile apparatus in high activating (HA) solution. Consistent with the characteristics of fast- vs. slow-twitch muscle, EDL and SOL fibers showed faster or slower transient kinetics, respectively. In EDL, caffeine-induced force (see Figure 1B) developed differentially with age in all genotypes. In wt single fibers, force amplitudes initially increased with age to significantly drop again in senile animals. In contrast, in the R349P desmin knock-in background, force developed oppositely in het fibers (decrease in the aged group and recovery to adult levels in the senile group) or did not vary significantly for hom fibers. Within age groups, we discovered isolated, genotype-specific significant differences that were, however, not systematic (see Figure 1B). Unlike caffeine-induced force, maximum Ca${}^{2+}$-saturated force was unchanged in EDL single fibers, regardless of age or genotype (Figure 1C). The ratio of caffeine-induced to maximum force amplitudes serves as an indicator of SR Ca${}^{2+}$ filling, and showed a similar behavior as the former (see Figure 1D). While maximum force amplitudes in SOL single fibers were generally similar to those in EDL fibers (see Figure 1C), caffeine-induced peak force levels were roughly two times smaller (see Figure 1B). Within SOL fibers, no difference among genotypes was seen, while age had a strong negative effect on force amplitudes, which were significantly reduced in wt preparations through age, and in het/hom fibers between the adult and the senile age group. Maximum attainable force levels were also impeded by age and displayed a significant decline during aging within each genotype. Particularly hom fibers were already significantly reduced in the adult age cohort, while the still better performing wt and het fibers gradually declined to the level of hom fibers with further age. The combined differences regarding force ratios were restricted to a significant age-related, genotype-specific decline (Figure 1D).

**Figure 2:**
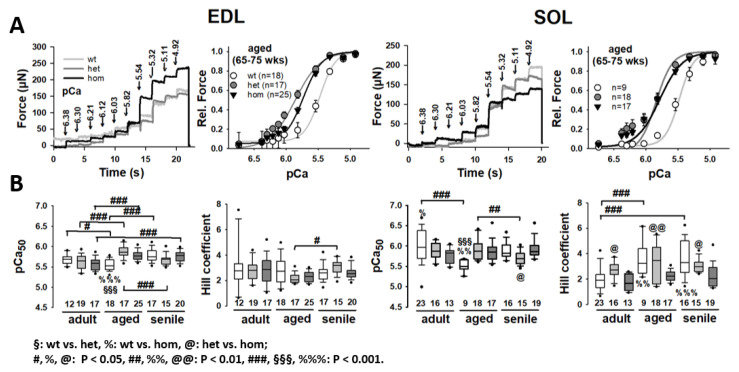
Ca${}^{2+}$ sensitivity of the contractile apparatus in single EDL and SOL fibers from adult, aged, and senile R349P desmin mice. (**A**) Representative force recordings in an aged single EDL (left) and SOL (right) fiber for each genotype showing increasing force for each pCa step change. The mean pCa–force curves along with the mean reconstructed Hill fit to the data are shown to the right. The curves display a marked left-shift in the R349P desmin knock-in background. Group analysis of pCa${}_{50}$ values and Hill coefficients in panel (**B**) show a significantly increased Ca${}^{2+}$ sensitivity in aged R349P desmin knock-in animals over the wt which is caught up in the senile group. Likewise, in the adult age group, Ca${}^{2+}$ sensitivity is similar between genotypes. In EDL, there is a significant trend towards increasing Ca${}^{2+}$ sensitivity in the R349P desmin knock-in background with age, while in SOL, significant age-related changes were only observed in the wt. Overall, differences between wt and hom preparations became more distinct with age. Significance tested with two-way ANOVA followed by post hoc analysis (Bonferroni). Numbers in box plots: number of single fibers analyzed.

To elaborate on the Ca${}^{2+}$ sensitivity of single fibers carrying the desmin R349P mutation, force–pCa recordings were performed across all age groups in EDL and SOL muscles. Figure 2A (top left) shows representative force–pCa curves from each genotype in aged animals. To the right, the respective average force–pCa of this age group is displayed for EDL (left) and SOL (right), along with the average reconstructed Hill fits (see Figure 2B). The curves in Figure 2A already suggest a marked left-shift of the sensor curve in R349P desmin knock-in single fibers over wt, indicating a myofibrillar Ca${}^{2+}$ sensitization in presence of mutant desmin. This was confirmed in the group analysis, where adult hom R349P desmin knock-in EDL single fibers were initially less Ca${}^{2+}$ sensitive but became more sensitive than the wt in aged animals. This also agrees with wt single EDL fibers reaching their largest pCa${}_{50}$ value one age bin later than hom fibers. Within the oldest age cohort (senile), all pCa${}_{50}$ values had finally reached similar levels among genotypes. Unlike EDL, SOL only displayed age-related effects in the wt, with an initial Ca${}^{2+}$-desensitization (from the adult to aged animals) that was later revoked in senile animals. Like the EDL, adult hom SOL fibers showed yet significantly depressed pCa${}_{50}$ values, which, however, strongly increased in the aged age cohort while wt fibers only matched those high levels in the senile age group (see Figure 2B). Het fibers showed similar trends as hom fibers, yet did not reach statistical significance.

The Hill coefficients in EDL single fibers showed no significant differences regarding genotypes. Age, however, had a significant influence on het fibers between aged and senile animals. In SOL single fibers, differences were present among genotypes, with lower coefficient values for fibers expressing the R349P mutation (except het adult). Again, age had a strong influence on wt fibers, leading to a significant increase in the Hill coefficient in aged and senile fibers over adult fibers, indicating a higher Ca${}^{2+}$ cooperativity of the myofibrillar Ca${}^{2+}$–biosensor complex.

### 2.2. Steady-State RLT Curves Demonstrate a Decreased Axial Compliance in R349P Desmin Knock-in Single Fibers

**Figure 3:**
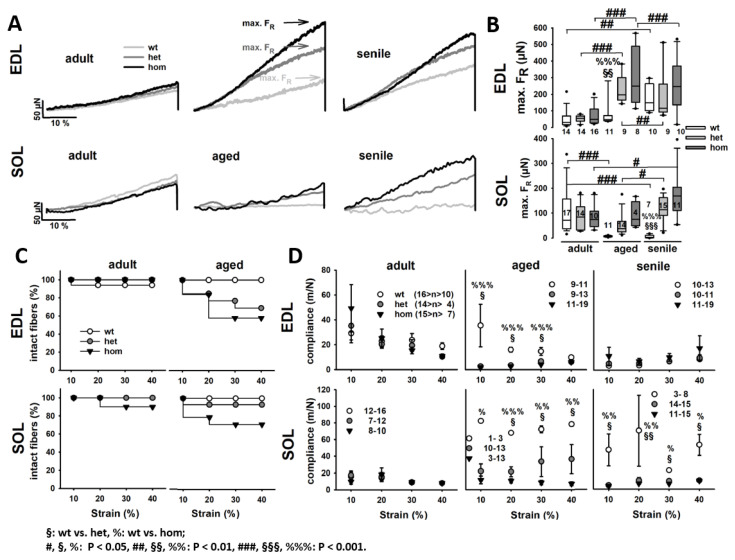
Passive steady-state RLT curves of single EDL and SOL fiber segments from adult, aged, and senile mice carrying the R349P desmin mutation. (**A**) Representative force recordings in single EDL (top) and SOL (bottom) fibers for each genotype and age cohort investigated. During aging, het and hom R349P desmin knock-in fibers present with a markedly steeper curve and increased maximum restoration forces. This was confirmed in the group analysis in (**B**), showing a significantly increased restoration force in both het and hom fibers, already in the aged mice. Force values in wt fibers remained reduced, but eventually increased within the senile age group. (**C**) Kaplan–Meier survival plots, shown for the adult and aged group, depict a much lower survival of mutant single fibers during the stretch protocol compared to wt fibers. (**D**) Axial compliance derived from slopes to the RLT curves to each section of 10% stretch decreases with stretch. Mutant fibers generally show lower compliance values than wt fibers, except for adult mice in both EDL and SOL, and senile mice in EDL. Significance tested with two-way ANOVA followed by post hoc analysis (Bonferroni). Numbers in box plots: number of single fibers analyzed.

Figure 3A shows a series of example RLT curves from single fibers of each genotype and age group from EDL and SOL muscles. The example traces already suggest that the RLT slope strongly increases with age in single fibers with mutation background, more so in EDL over SOL muscle. This increase occurred in a less-pronounced fashion in wt EDL single fibers, while it was absent in wt SOL samples. These remained at similar levels independent of age. As a measure for steady-state stiffness at 140% L${}_{0}$, maximum restoration force (max. F${}_{R}$) was analyzed in Figure 3B, statistically confirming the behavior seen in the examples. In the adult age group, max. F${}_{R}$ values were all similar between genotypes. While max. F${}_{R}$ increased in all EDL single fibers with age, it did so even stronger and earlier in the R349P knock-in background, significantly exceeding the wt in the aged group. At the senile age, the wt had then caught up with the mutants. Although not significant, het fibers showed smaller max. F${}_{R}$ values than hom fibers. This trend was also seen in SOL fibers, except for wt fibers showing significantly decreased max. F${}_{R}$ values with age in comparison to the adult group. The higher max. F${}_{R}$ values in the R349P knock-in background also impacted on a lower survival of single fibers during stretch. In both EDL and SOL muscles, mutant single fibers already broke at lower strains compared to the wt, while fibers heterozygous for R349P displayed a better survival than hom fibers (see Figure 3C). As these results indicate an increased axial stiffness, Figure 3D summarizes the analysis of the 10% strain-wise calculated axial compliance. For adult samples of EDL and SOL, axial compliance was similar for all genotypes. Yet, in the aged EDL cohort, compliance of mutant fibers was already significantly reduced. For even older animals (EDL senile), the wt then declined to similar low compliances as the mutants for EDL muscle fibers, whereas for SOL, compliance remained at high levels and even seemed to increase further with age in the wt.

### 2.3. Axial Viscosity Is Unaltered by the R349P Mutation in Single EDL and SOL Fibers

**Figure 4:**
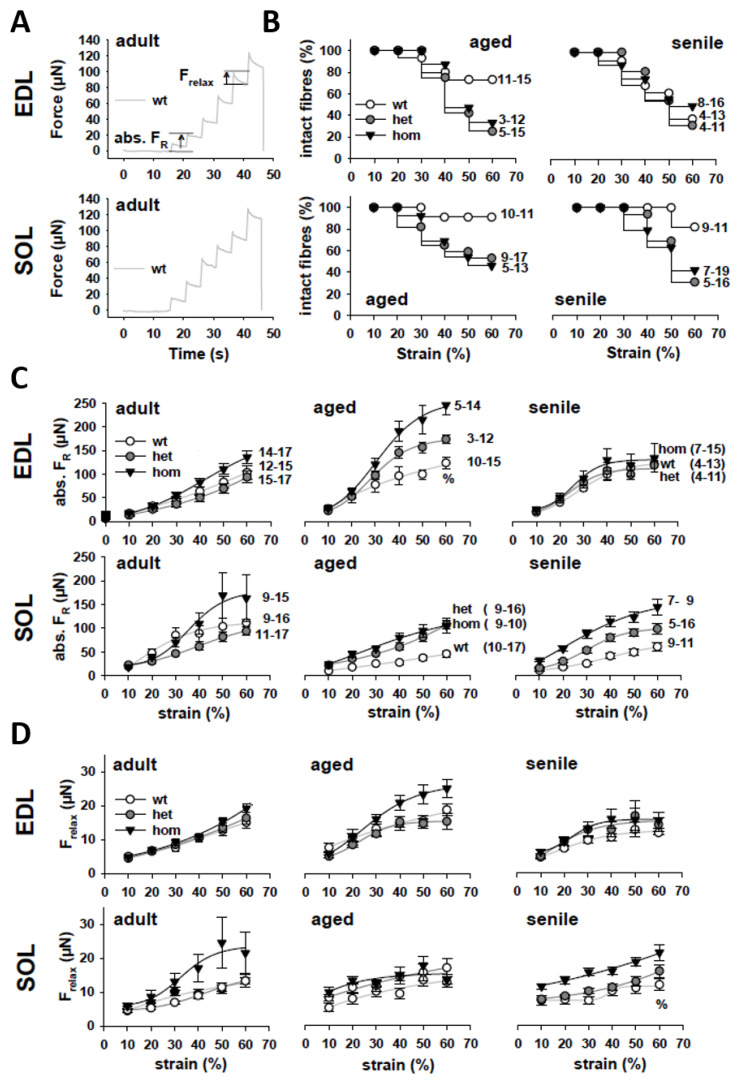
Visco-elastic behavior of single muscle fibers from EDL and SOL muscle carrying the R349P desmin mutation during aging. (**A**) Representative examples of quick stretch-jump experiments, stretching adult EDL and SOL fibers in 10% bins to 160% L${}_{0}$. (**B**) Kaplan–Meier survival plots demonstrate a worsened stretch resistance of mutant fibers. (**C**) Group analysis of F${}_{R}$ across ages in EDL (top) and SOL (bottom) fibers shows increased absolute restoration force levels in mutants over wt fibers for almost all ages and in both muscles. (**D**) Force relaxation amplitudes with stretch suggest almost similar viscous relaxation with a tendency for higher viscous relaxation in mutant fibers over the wt. Significance tested with two-way ANOVA followed by post hoc analysis (Bonferroni). Error bars: standard error.

To assess axial viscosity in single muscle fibers, we carried out ultra-fast stretch jumps as shown in Figure 4A for wt adult single fibers from EDL and SOL muscle. Each new stretch jump was answered by an instantaneous restoration force (F${}_{R}$) increase to a maximum, followed by viscous relaxation (F${}_{relax}$) to a new steady-state level during the holding phase. In compliance with findings from RLT recordings, mutant single fibers had a much higher chance of rupture during these strenuous sudden stretches as compared to wt fibers (see Figure 4B). Analysis of maximum F${}_{R}$ amplitudes with stretch, reflecting axial stiffness, matches findings from RLT curves and suggests higher restoration forces in mutant single fibers (see Figure 4C). However, relaxation force F${}_{relax}$ (difference between maximum F${}_{R}$ and steady-state F${}_{R}$ within the same stretch jump) was not significantly different between either genotype or ages (see Figure 4D), arguing against any involvement of the R349P mutant desmin in titin-related viscous relaxation processes.

### 2.4. Fast Phase of Unloaded Speed of Shortening Is Accelerated Particularly in Aged Het R349P Desmin Single Fibers and Even Speeds up with Age

**Figure 5:**
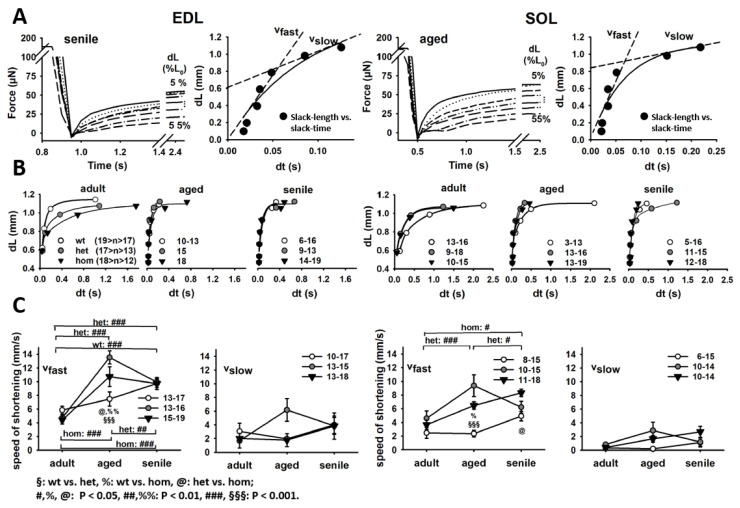
The fast phase of unloaded speed of shortening in single EDL and SOL fiber segments from R349P desmin mice is markedly increased in aged het fibers. (**A**) Representative slack-test of a single senile EDL (left) and aged SOL (right) fiber. The “slack time” was extracted for each “slack length” and the dL–dt relationship plotted in the right subpanels along with a biexponential fit and a linear velocity approximation in the lower dL (fast) and upper dL (slow) regime. (**B**) Group analyses of all single fibers from each genotype and age described by biexponential fit curves. The group analysis of the linear fast (v${}_{fast}$) and slow (v${}_{slow}$) phase for all fibers of each available genotype and muscle is shown in (**C**). Fast shortening speed gradually increased in wt fibers with age. Het samples perform the fastest in the aged fiber cohort. In hom fibers, shortening speeds also increase with age and only display a single decline for senile EDL muscle. Significance tested with two-way ANOVA followed by post-hoc analysis (Bonferroni). Numbers next to symbol legends: number of single fibers analyzed.

The observed increased passive stiffness in R349P mutant desmin single fibers suggests a negative influence on muscle contraction kinetics, e.g., unloaded speed of shortening. To address this question, we performed so-called slack-tests. Figure 5A shows representative recordings of a senile EDL (left) and an aged SOL (right) single fiber. After reaching steady-state maximum isometric contraction in HA solution, the VC quickly introduced a slack of defined length dL to the fiber. Consequently, force dropped to zero and redeveloped over time (dt). The relation dL vs. dt is plotted to the right in Figure 5A; also shown are the linearly derived fast and slow velocities v${}_{fast}$ and v${}_{slow}$ from the respective section of the double exponential fit. Figure 5B shows the dL-dt plots for all age groups and genotypes for both muscles, and Figure 5C shows the statistical analysis of v${}_{fast}$ and v${}_{slow}$. v${}_{fast}$ reflects the initial, unloaded phase, whereas v${}_{slow}$ represents the internally loaded phase that occurs while taking up larger “slack lengths” ([22]). Notably, v${}_{fast}$ increased with age in all genotypes, while it decreased again in senile mutation-bearing fibers, except for hom SOL fibers. In this context, it was even more compelling that mutant fibers performed significantly faster than wt fibers in aged animals. Although v${}_{slow}$ qualitatively showed a similar trend, there were no statistical significances regarding age or genotype.

## 3. Discussion

Desminopathies comprise a heterogeneous group of inherited and sporadic myopathies which, in most cases, share a common morphological picture comprising sarcoplasmic and subsarcolemmal desmin-positive protein aggregates and signs of myofibrillar degeneration [14,16,23]. In general, analyses of the pathophysiology of human desminopathies are hampered by the very limited amount of available human muscle tissue and the fact that alterations noticed in diagnostic muscle biopsies nearly always reflect late stages of the disease. To overcome these limitations, we generated the patient-mimicking R349P desmin knock-in desminopathy mouse model, which harbors the orthologs of the most frequent human desmin mutation R350P [18]. This mouse line has already proven invaluable in performing age-related morphometric analyses of cytoarchitectural changes in early disease stages in single fibers from slow- and fast-twitch muscles using multiphoton Second Harmonic Generation (SHG) microscopy [19]. In that study, we showed a pre-aged morphological phenotype depicting sarcomeric lattice disorder and myofibrillar angular distribution in both EDL and SOL single fibers [19]. On a single-fiber level, such distorted myofibrillar cytoarchitecture is already a structural determinant of muscle weakness per se, as the resulting force vector is smaller compared to if all myofibrils were perfectly aligned [24,25,26]. For human R350P desminopathy, apart from clinical assessment of overall force in proximal and distal muscle groups according to MRC grades [14], no information on active force production on the sub-organ level (single fibers, fiber bundles) is available. For the murine R349P desmin knock-in model, initial characterization of small SOL fiber bundles at preclinical stages in young mice [18], as well as a very recent whole age-dependent study of ours on small EDL and SOL fiber bundles from 17 to >60 weeks of age, documented a pre-aged increase in passive axial stiffness. Yet, due to an observed increased extracellular fibrosis in aged R349P desmin knock-in muscles [19], the possibility remains that stiffer inter-fiber elastic elements (e.g., ECM) are responsible for the reduced axial compliance in small EDL and SOL fiber bundles. To asses this question, we eliminated influence of ECM components on biomechanics recordings by advancing to isolated single muscle fiber preparations. The advantage of dissected single fibers not containing ECM connections to surrounding elements, i.e., being void of neighboring fibers, provides a pure preparation to exclusively focus on the effect of mutated desmin on cytoskeletal axial fiber biomechanics.

### 3.1. Mutant R349P Desmin Does Not Affect Single Fiber Active Biomechanics in Either Fast- or Slow-Twitch Muscles, While Age Weakened Fibers of Wt Animals

Similar to previous studies in fiber bundles [19,21], we observe that the R349P desminopathy seemed to leave active contractile properties in single muscle fibers largely unaffected. This is particularly true for caffeine-induced Ca${}^{2+}$-mediated force transients, which only revealed some unsystematic genotype-related differences in EDL samples. If anything, maximum force generation is significantly reduced in adult SOL single fibers, which was also reported in the literature [27], but in our case subsided with age. In general, effects of age in forms of declining force production were more prominent in SOL wt fibers and were also observed in wt EDL fibers. Detailed systematic age-related studies on contractile properties in fast- and slow-twitch muscle are rare or mostly focus on whole muscle. In 2-year-old versus 6-mo-old rats, twitch and tetanic force were lower in elderly animals, but no differences in maximum force-generating capacity were found in either slow- or fast-twitch muscles at either age [28]. In these muscles however, a decline in absolute isometric tetanic force to ∼75% from young (2–3 mo) to adult (9–10 mo) to aged (26–27 mo) mice was reported for both EDL and SOL. This difference prevailed after normalization to specific tetanic force for fast-twitch EDL, while age effects vanished for SOL after normalization [29]. Last, an age-related study in dystrophic *mdx* mice reported no difference when comparing single skinned SOL and EDL fibers from young (3–6 weeks) and adult (17–23 weeks) animals [30]. The recognition of considerable variability in (specific) isometric force values between study groups has been stated to render comparisons between whole muscles, fiber bundles and single fibers with respect to aging difficult [29].

Unfortunately, when this study was initiated, our *MyoRobot* system was not yet equipped with an optics system to measure the fiber diameter to normalize forces to cross-sectional area (also known as specific force or stress). Yet, the absolute single-fiber force levels presented here are well in the range of those reported by Stelzer et al. (2003) [31] in SOL fibers from adult (8–12 weeks) mice, ∼150 μN per fiber at maximum Ca${}^{2+}$ activation. Furthermore, assuming a fiber diameter of 30–40 μm and circular cross section, based on a study by Diermeier et al. (2017) [19], the measured specific force values compute approximately to 15 N/cm${}^{2}$ or 150 kPa, which is in agreement with single-fiber specific force values from literature [30,31].

Regarding the Ca${}^{2+}$ sensitivity of the contractile apparatus, a pre-aged phenotype in the R349P background was observed. Particularly mutation-bearing EDL fibers displayed a myofibrillar Ca${}^{2+}$ sensitization already within the aged age group, while in wt littermates, this became only apparent in the senile group. This corroborates well with results from our recent age-related biomechanical assessment of R349P desmin small fiber bundles, where a similar desensitization of ∼0.2–0.3 pCa units was seen from the adult to the aged age group in mutant EDL bundles [21]. For SOL, the data here do not seem to confirm a consistent trend among genotypes with age, apart from a large scattering between individual SOL fibers. This could be due to marked differences of pCa${}_{50}$ values between fast- and slow-twitch fibers being present in the SOL muscle, as it contains an almost equal proportion of either fiber type [32,33]. Unlike in previous studies regarding single fiber Ca${}^{2+}$ sensitivity assessment (see, e.g., in [33]), we did not attempt to type fibers for myosin heavy chain (MHC) isoforms for technical reasons, and thus this may at least partially explain the observed variability. However, from our previous work assessing MHC composition in SOL muscle homogenates for all three genotypes, we are confident that hom fibers present with higher slow-type MHC I content over wt and het fibers [19]. Therefore, the large variability towards higher pCa${}_{50}$ values in aged and senile single SOL fibers (see Figure 2B) is in good agreement with the presence of higher pCa${}_{50}$ values in type I over type II fibers [33]. Moreover, the absolute pCa${}_{50}$ values presented here are in good agreement with the aforementioned study [33].

### 3.2. Passive Axial Biomechanics Is Shifted Towards a Pre-Aged Stiffer Phenotype in Single Fast-Twitch Fibers by R349P Desmin

In compliance with our previous assessment in small fiber bundles [18,19,21], mutated single fibers showed a marked increase in passive restoration forces, which was particularly visible in aged and senile muscle fibers with R349P background. While restoration forces in adult animals were at a similar level, aged het and hom single EDL fibers already displayed as large force values as only found in the wt senile group. A fiber stiffening with age was likewise reported in human *vastus lateralis* [34]. Thus, our observed enlarged stiffness in adult mutant single fibers points towards a pre-aged phenotype in fast-twitch muscle. Matching with our findings in EDL fiber bundles [21], increasing restoration force and decreasing compliance were already detected at younger ages here, and were more pronounced in hom R349P desmin knock-in mice [21].

Analysis of compliance in SOL single fibers also shows an augmented passive axial stiffness in het and hom R349P desmin knock-in single fibers. However, in contrast to results from EDL single fibers, it is the wt becoming more compliant with age. Although mutant SOL single fibers are significantly stiffer than their wt counterparts, which was also reported in literature [6], their compliance in fibers from aged and senile animals, ranging from 50 to 80 m/N, is more than two-fold higher than in EDL fibers. This is in contradiction with literature data suggesting enlarged stiffness in slow-twitch over fast-twitch muscle [35] and may be attributed to the small experiment numbers (*n* < 3 for aged, *n* < 8 for senile) and large data scattering in these age cohorts (please note that due to constraints on breeding colonies, availability of aged and senile animals was limited). Previously, it was reported that the loss of desmin results in fiber stiffening, which was found unrelated to alterations in titin [36]. In the R349P desminopathy model, we likewise observed that the lacking functional desmin causes a stiffening in mutant fibers. As titin was suggested to play a minor role in this process and a comparison of our results to fiber bundles including ECM [21] revealed a similar fiber stiffening, we assume desmin to be part of the three-component stiffness model introduced by Anderson et al. (2002) [37]. The model concludes that tension is equally distributed on each element, but each element has its own extension [37]. In such a configuration, the loss of functional desmin would result in an increased axial stiffness within mutated fibers and would explain the R349P desmin-related stiffening.

A direct comparison of bundles (compliance up to 10 m/N, [21]) and single fibers (compliance up to 80 m/N) suggests that ECM components in fiber bundles likely introduce additional passive stiffness, e.g., through inter-fiber connections. Alternatively, larger cross-sectional area in bundles carrying the R349P mutation may be dissipating restoration forces between both intracellular (i.e., mutated desmin) and extracellular non-contractile elements. Although an increase in ECM collagen was detected in our previous study in hom R349P SOL bundles over het bundles [19], a contribution of other ECM components cannot be ruled out and deserves further investigation. Nevertheless, our *MyoRobot* approach was able to extend our previous knowledge on R349P axial muscle stiffness to single fibers and also to include a larger age range, which unravelled aging effects in normal muscle as well. For instance, compliance values in wt EDL single-fiber preparations remained mostly stationary (up to the aged group) and only declined in old animals (senile group), while SOL fibers became more compliant (less stiff) with age. This is in agreement with a comparative study on murine *tibialis anterior* single muscle fibers and small fiber bundles, where single fibers from old mice showed a tendency towards reduced elasticity moduli (reflecting smaller stiffness/larger compliance values) [38]. Moreover, the researchers showed that the intrinsic stiffness of ECM increased with age, which was indicated by larger Young moduli in fiber bundles over single fibers, and in particular, a two-fold increased bundle stiffness in old versus adult *tibialis anterior* fiber bundles [38], which was similarly investigated in human *vastus lateralis* bundles [34].

A similarly increased modulus (quadratic modulus, kP/μm${}^{2}$) was shown in EDL fiber bundles over single fibers from young (7–9 weeks) wt mice [39]. When comparing our axial single fiber compliance to the corresponding values in small fiber bundles of our associated study (i.e., SOL ∼1–4 m/N and EDL ∼1–6 m/N [21]), our single fibers consistently display a higher compliance, reflecting enlarged stiffness in bundles over single fibers. Meyer et al. (2011) [39] also provide an elegant experimental explanation for the increased stiffness in fiber bundles over single fibers, in that ECM contribution to nonlinear bundle stiffness is set out by spreading the sarcomere length distribution of individual fibers within the bundle. This superposes different RLT curves from single fibers in a bundle to a non-linear resulting stiffness behavior. This is most probably due to different lateral and axial forces acting on adjacent single fibers through ECM-mediated focal adhesion connections, i.e., integrins [40]. It is of note that the absolute axial stiffness (compliance) in our study and those aforementioned ones cannot be directly compared, as different methods were employed, and our system could not yet assess single fiber cross-sectional area and sarcomere length distributions as in later development stages of our system [41].

### 3.3. Unloaded Speed of Shortening Suggests Faster Contractions of R349P Desmin Knock-in Single Fibers

With our implementation of a VC actuator within the *MyoRobot* [22], it was also possible to address whether the increased axial stiffness in single fibers in the R349P desmin knock-in background would impact on unloaded shortening, given the fact that the isometric maximum force development was rather unaffected. Absolute velocities for the fast phase of contraction ranged from 4 mm/s to 12 mm/s for EDL single fibers and 2–6 mm/s for SOL fibers. These findings are well in the range of velocities reported for single EDL fibers from wt mice unrelated to this study [22]. This demonstrates the robustness of our automated biomechatronics system to assess active biomechanical properties in single fibers across studies and organ scales. Similar to our previous study in small fiber bundles, fast velocities gradually increased with age, particularly in het mice [21]. However, in the aforementioned study, only few numbers of observations were available for EDL muscle bundles, which complicates a robust comparison. Rather, for SOL bundles with higher experiment numbers, fast velocities showed a tendency for slowed down shortening in young R349P knock-in mice that was abrogated in the adult age group. Intriguingly, just as in our single-fiber recordings, het fiber bundles were the fastest in the aged cohort [21]. Although single fibers reflect a purer preparation void of ECM components, it is unlikely that this sole difference impedes on contractile shortening, but an uneven distribution of single fiber sarcomere lengths within a fiber bundle may also add to this effect. Yet again, at that time, our *MyoRobot* system was not yet equipped with an optics system to assess sarcomere length distributions.

In literature, age-related studies regarding unloaded shortening in single fibers are scarce. One of the few studies on rat EDL single fibers found an unchanged maximum shortening velocity in adult (9 mo) versus senescent (30 mo) animals, whereas SOL fibers from old rats [42] were slower. In male human *vastus lateralis* skinned single fibers, shortening velocities were reduced in type IIA fibers but not type I fibers, while the opposite was found for women [43]. For murine muscles, a detailed sex- and age-related study is not available, to our knowledge. The reason for the increased speed of shortening, particularly visible in het R349P desminopathy muscle fibers, cannot unambiguously be explained at current, especially considering our recent finding that slow-type MHC I isoforms were upregulated in R349P desmin knock-in muscles, while fast-twitch MHC II isoforms were downregulated [19]. Thus, we suspect R349P mutated desmin to have some influence on the kinetics of weak cross-bridge attachment that was reported as a key factor for unloaded speed of shortening [44]. Whether this may be an explanation for the increased shortening velocity in desminopathy single fibers deserves future investigation.

## 4. Materials and Methods

### 4.1. Mouse Model—R349P Desmin Knock-in Mouse

Heterozygous (het) and homozygous (hom) littermates of the R349P desmin knock-in mouse model B6J.129Sv-Des${}^{tm1.1Ccrs}$ (http://www.informatics.jax.org/allele/ MGI:5708562) [18,45] were used. Littermates not carrying the R349P desmin mutation served as wild type (wt) control. Here, we extended our previous biomechanics study on small fiber bundles from only young mutant mice [19] towards three older age groups, spanning 35–45 weeks (adult), 65–75 weeks (aged), and 90–96 weeks (senile). All animal-related work was performed in accordance with the German Animal Welfare Act (Tierschutzgesetz), as well as the German Regulations for the protection of animals used for experimental purposes or other scientific purposes (Tierschutz-Versuchstierverordnung). The governmental Office for Animal Care and Use (Regierung von Mittelfranken, 91511 Ansbach, Germany; reference number TS-14/2015) approved the investigations. All applicable international, national, and institutional guidelines for the care and use of animals were followed.

### 4.2. Chemical Solutions

All muscle dissection was performed in Krebs solution containing (mM) 120 NaCl, 4.7 KCl, 1.2 KH${}_{2}$PO${}_{4}$, 1.2 MgSO${}_{4}$x7H${}_{2}$O, 24.8 NaHCO${}_{3}$, 0.1 M glucose, and 0.1% FCS (FBS), pH 7.3. A Ca${}^{2+}$-free, high K${}^{+}$ solution (HKS) was used to permanently depolarize the muscle cell membrane to abolish excitability during manual tethering of fascicles and isolation of single fiber segments. HKS contained 140 K-glutamate, 10 Hepes, 10 glucose, 10 MgCl${}_{2}$, and 1 EGTA (ethylene glycol-bis(β-aminoethyl ether)-N,N,N’,N’-tetraacetic acid), pH 7.0. To maximally Ca${}^{2+}$-activate single fibers, a Ca${}^{2+}$-saturated high activating internal solution (HA) was used containing 30 Hepes, 6.05 Mg(OH)${}_{2}$, 30 EGTA, 29 CaCO${}_{3}$, 8 Na${}_{2}$ATP, and 10 Na${}_{2}$CP, pH 7.2. Free Ca${}^{2+}$ of HA was calculated to ∼12.5 μM using the chelator–ligand binding software React (developed by Geoffrey Lee, University of Glasgow). To maximally relax single fibers and to completely buffer Ca${}^{2+}$ ions each time a fiber was exposed to Ca${}^{2+}$, high relaxing solution (HR) was used that had the same composition as HA except for not containing any Ca${}^{2+}$ (for practical reasons of pCa calculations, a pCa of 9 is assumed in HR). Mixtures of HA and HR were calculated to obtain a given pCa of the internal solution for graded Ca${}^{2+}$-activation in pCa–force response curves using React and consisted of HA:HR ratios of 0.3:0.7, 0.5:0.5, 0.55:0.45, 0.6:0.4, 0.65:0.35, 0.7:0.3, 0.8:0.2, 0.9:0.1, 0.95:0.05, 0.98:0.02, and 1:0, converting to pCa values of 6.74, 6.38, 6.30, 6.21, 6.12, 6.03, 5.82, 5.54, 5.32, 5.11, and 4.92, respectively. Low relaxing solution (LR) served as an intermediate step after HR or loading solution (LS, see below) to replace the high affinity Ca${}^{2+}$ chelator EGTA for low affinity HDTA (1,6-diaminohexane-N,N,N’,N’-tetraacetic acid). LR contained 30 Hepes, 7.86 Mg(OH)${}_{2}$, 87.8 K-glutamate, 6.6 HDTA, 0.4 EGTA, 8 Na${}_{2}$ATP, and 10 Na${}_{2}$CP (creatine phosphate), pH 7.2. LS was a mixture of HA and HR titrated to a free Ca${}^{2+}$ of ∼300 nM to reload the sarcoplasmic reticulum for defined incubation times. RS served as release solution for Ca${}^{2+}$ ions from the SR and was LR supplemented with 30 mM caffeine. All solutions were thawed from stocks at the day of experiments and freshly supplemented with creatine kinase (CK, Sigma-Aldrich/Merck KGaA, Darmstadt, Germany) to ∼300 U/ml or ∼3 U/well and sodium azide (0.1 M NaN${}_{3}$), the latter to prevent mitochondrial Ca${}^{2+}$ uptake ([46]). To initially chemically permeabilize a single fiber, saponin was added to HR in a separate well of the *MyoRobot* rack to a concentration of 0.1% (*w*/*v*).

### 4.3. Preparation of Single Muscle Fibers

Mice were anesthetized via isoflurane inhalation and sacrificed by cervical dislocation. The hind limbs were cut off and transferred to Krebs solution. SOL and EDL muscles were dissected under a stereo microscope (Olympus SZX7, Olympus, Hamburg, Germany), while being pinned under slight stretch into a Sylgard^©^ (Dow Corning, Wiesbaden, Germany)-coated petri dish. Upon completing the dissection, Krebs solution was exchanged for HKS, allowing for 15 min equilibration, before single fibers were manually dissected with fine forceps.

### 4.4. Assessment of Active and Passive Biomechanics in Single Muscle Fibers in an Automated *MyoRobot* Environment

Biomechanics recordings were conducted using the *MyoRobot*, a novel automated biomechatronics system combining high-precision VC actuation with force sensor technology [22]. After isolation, the single-fiber segment (length at least 2 mm) was transferred to the *MyoRobot* multi-well rack in a custom-made Perspex chamber while submerged in HKS solution, placed below the pins of the FT and VC, and fixed to both pins via a tweezer mechanism. For details on the biomechatronics system and sensor and actuation implementation, please refer to the work in [22]. Every protocol started with a chemical permeabilization of single fibers in HR supplemented with saponin for 20 s. An automated set of biomechanical recordings on the same preparation was then executed, consisting of sequential runs of (i) caffeine-induced, Ca${}^{2+}$-mediated force generation, (ii) pCa–force curves, (iii) speed of shortening (slack test), (iv) passive stiffness—resting length–tension curve (RLT), and (v) assessment of visco-elastic passive behavior:

- **Caffeine-induced, Ca${}^{2+}$-mediated force generation:** After fiber permeabilization, the fiber was shortly dipped into HR to wash off remaining saponin and to buffer internal Ca${}^{2+}$. Subsequently, it was translocated to LR for 60 s, after which the SR was loaded in LS for 60 s. The caffeine-induced force transient was triggered by exposure to RS for 60 s, while maximum force was induced via HA solution for 5 s (see Figure 1).
- **Ca${}^{2+}$ sensitivity of the contractile apparatus, pCa–force curves:** The fiber was sequentially exposed to solutions of increasing Ca${}^{2+}$ ion concentrations (decreasing pCa values (−log${}_{10}$[Ca${}^{2+}$])) for a duration of 20 s (see Figure 2).
- **Unloaded speed of shortening (slack test):** The muscle fiber was held at resting length L${}_{0}$ and transferred to HA solution, resulting in maximum isometric contraction. Upon achieving steady-state force, the VC pin moved at maximum speed towards the FT, slacking the fiber by a defined percentage of L${}_{0}$ (5%, 10%, 20%, 30%, 40%, 50%, or 55%) as force dropped to 0 mN. While taking up the slack, force re-established in the presence of saturating Ca${}^{2+}$. Once the next force plateau was reached, the fiber was washed in HR to remove excessive Ca${}^{2+}$ and to relax the myofibrils before moving on to the next consecutive slack length. For this recording, sampling rate was set to 2 kHz (see Figure 3).
- **Passive stiffness—RLT curves:** To assess passive axial stiffness, the muscle fiber was kept in LR solution to avoid active contraction. The fiber was continuously stretched at a slow speed (0.44 μm/s) to 140% of L${}_{0}$ (L${}_{0}$∼1950 μm) by moving the actuator pin away from the FT pin. Restoration force was continuously recorded. To every 10% stretch bin, a linear fit was applied to calculate the fiber’s compliance, reflected by the inverse of that slope, and thus the inverse of stiffness (see Figure 4).
- **Visco-elastic passive behavior:** To assess the visco-elastic passive behavior, the fiber was stretched in a sudden staircase-like pattern in 10% L${}_{0}$ steps to 160% L${}_{0}$ with a holding time of 10 s. To prevent any active contraction, the fiber was kept in LR during the recording. The force response of the fiber comprised of an instantaneous passive restoration force and a force relaxation, with an exponential decay of force back to a steady-state level (see Figure 5).

### 4.5. Data Analysis and Statistics

*MyoRobot* data were processed with analysis protocols in RStudio (RStudio Inc., rstudio.com, Boston, Massachusetts, USA) while plotted and statistically evaluated with SigmaPlot (Systat Software Inc., sigmaplot.co.uk, San Jose, California, USA). All data traces were smoothed with a moving average filter. For pCa curves, the plateau force close to the end of each pCa step was determined by the software and plotted against the corresponding pCa value. The scatter plot of normalized force (normalized to max. force at pCa 4.92) was fitted to a four-parameter Hill equation ($y=y_{0}+\frac{a*10^{-bx}}{c^{b}+10^{-bx}}$) utilizing least-square methods with the physiological constraints y${}_{0}$ = 0 and a = 1. The steepness (b, Hill coefficient) and the deflection point ($-log_{10}\left(\left[Ca^{2+}\right]\right)$, pCa${}_{50}$) of every individual curve fit were used to reconstruct a mean fit to the averaged data points (see Figure 2). For speed of shortening (slack tests), a 5% threshold criterion was established from the maximum isometric force of the first slack. This threshold defined significant “force redevelopment” for this and all consecutive slack lengths (dL). The time needed to cross this force threshold was called slack time (dt) and was plotted against the respective slack length dL. The resulting dt–dL scatter plot was fitted with a bi-exponential function ($y=a\left(1-e^{\kappa 1+dt}\right)+c\left(1-e^{\kappa 2+dt}\right)$). Its derivative represented the nonlinear slack length-dependent shortening velocity v(dL). The dL–dt range was divided in a fast (unloaded phase, <45% slack length) and a slow phase (internally loaded phase, >45% slack length) as described in [22]. Passive stiffness—RLT curves: To every 10% L${}_{0}$ stretch bin, a linear fit was applied and the respective increase/steepness computed to obtain axial stiffness and compliance (inverse increase). Visco-elastic behavior: The force baseline (F${}_{0}$) was determined as the last 5 s before the first step while absolute restoration force ($F_{abs}=max_{n*10\%}-F_{0}$) of each 10% stretch step was calculated as the difference of maximum recorded force of the corresponding step to the baseline. Force relaxation was obtained from the difference between maximum and minimum force recorded within the same step ($F_{relax}=max_{n*10\%}-min_{n*10\%}$). Statistical significance was assessed after probing for normality by applying two-way ANOVA tests (age bins and genotypes as variables), following post hoc analysis (Bonferroni) in SigmaPlot (ANOVA on ranks if normality was violated). Significance levels of *p* < 0.05 were considered significant, <0.01 and <0.001 considered strongly and highly significant, respectively. Significance levels involving age effects were depicted as #, while genotype differences were depicted as §: wt vs. het, %: wt vs. hom, and @: het vs. hom, respectively.

## 5. Conclusions

Our results confirm an increased passive steady-state stiffness in R349P desminopathy skeletal muscle. Our experiments on single muscle fibers, void of ECM, suggest that compromised biomechanics properties in the R349P desminopathy do not only originate from increased fibrosis, but also from mutant desmin inflicted damage to the cytoskeleton. Particularly in fast-twitch fibers, this results in a pre-aged, stiffened phenotype. Apart from a yet unexplained acceleration of speed of shortening, especially pronounced in aged het fibers, Ca${}^{2+}$-mediated active force was only mildly affected, if at all. Our *MyoRobot* system allows a highly versatile and modular design of automated execution of various additional muscle test protocols, e.g., eccentric contractions, that shall be of great value to the community to facilitate future myopathy and mechanistic studies related to skeletal muscle and aging.

## Abbreviations

The following abbreviations are used in this manuscript.

ECM	extracellular matrix
EDL	*M. extensor digitorum longus*
EGTA	ethylene glycol-bis(-aminoethyl ether)-N N N’ N’-tetraacetic acid
FBS	fetal bovine serum
FCS	fetal calf serum
FT	force transducer
HA	high activating (solution)
HDTA	hexamethylenediaminetetraacetic acid
Hepes	4-(2-hydroxyethyl)-1-piperazineethanesulfonic acid
het	heterozygous
HKS	high potassium solution
hom	homozygous
HR	high relaxing (solution)
IF	intermediate filament
LR	low relaxing (solution)
MHC	myosin heavy chain
RLT	resting length-tension
SOL	*M. soleus*
SR	sarcoplasmic reticulum
VC	voice coil
wt	wild type
ULF	unit length filament
